# Normalized Cortisol Reactivity Predicts Future Neuropsychological Functioning in Children With Mild/Moderate Asthma

**DOI:** 10.3389/fpsyg.2019.02570

**Published:** 2019-11-19

**Authors:** Sarah M. Dinces, Lauren N. Rowell, Jennifer Benson, Sarah N. Hile, Akaysha C. Tang, Robert D. Annett

**Affiliations:** ^1^Department of Psychology, The University of New Mexico, Albuquerque, NM, United States; ^2^Faculty of Education, The University of Hong Kong, Pok Fu Lam, Hong Kong; ^3^Department of Pediatrics, University of Mississippi Medical Center, Jackson, MS, United States

**Keywords:** normalized cortisol, HPA response, children, attention, memory, asthma

## Abstract

Cortisol reactivity to adrenocorticotropic hormone (ACTH) has been associated with neuropsychological processes including attention and memory in children with asthma. While cortisol reactivity to a psychological stressor is often considered a measure of current neuroendocrine functioning, this study examines the association of the cortisol reactivity and subsequent neuropsychological functioning. Using prospective data from the Childhood Asthma Management Program (CAMP), we explored the predictive ability of cortisol reactivity to ACTH and children’s later attention and memory using traditional and an alternative cortisol reactivity (normalized cortisol) measures. Cortisol reactivity was assessed at study entry and 1-year follow-up, and neuropsychological functioning was assessed at 3-year follow-up. Cortisol reactivity was assessed through plasma cortisol concentrations collected at baseline (CORT_BASELINE_) and 30 min post-ACTH challenge (CORT_POST–A__CTH_). An alternative measure of cortisol reactivity was developed through post-ACTH stimulation cortisol, normalized by cortisol by baseline (CORT_NORM__–ACTH_). CORT_*B*__ASELINE_ positively predicted year 3 attention, while CORT_NORM__–ACTH_ negatively predicted attention, suggesting convergence of cortisol variables in prediction of neuropsychological function. Year 1 CORT_ACTH_ positively predicted child memory at year 3; Year 1 CORT_NORM–ACTH_ negatively predicted year 3 sustained attentions. These findings demonstrate that HPA reactivity, including the application of normalized cortisol reactivity, can predict subsequent neuropsychological functioning of children with mild to moderate asthma.

## Introduction

Dysregulation of the stress response system has been implicated in psychological disorders (Faravelli et al., 2012; Ising et al., 2012; Spijker and van Rossum, 2012; Staufenbiel et al., 2013; Yildirim and Derksen, 2013; Mansueto and Faravelli, 2017; Wielaard et al., 2018; Yirmiya et al., 2018; Ciufolini et al., 2019) and associated with neuropsychological functioning (Lupien et al., 2009; Scassellati et al., 2012; Forns et al., 2014). The stress response system is activated by a perceived threat. Threat perception (e.g., perception of physical danger, psychosocial stressors, low glucose levels, or physical illness) results in activation of cortical and subcortical brain networks that operate in different timeframes, some very quickly and others more slowly (Liddell et al., 2004). Cortical networks respond immediately, as they do not rely upon the increased production of hormones, while subcortical networks take time to activate because they send signals throughout the body, which stimulates increased production of stress hormones such as cortisol. Cortisol production in the adrenal glands is stimulated by the hypothalamus and pituitary gland, both located in the brain.

The hypothalamic-pituitary-adrenal axis is the primary mechanism through which the body responds to a threat to homeostasis (McEwen, 1998). In response to threat perception, a series of hormonal responses are triggered, ending in the production of cortisol which is down regulated via negative feedback from the hippocampus, the brain structure involved in learning and memory. The hippocampus contains a dense population of the two cortisol receptors, mineralocorticoid receptors (MR) and glucocorticoid receptors (GR). Cortisol preferentially binds to MR under resting conditions, yet when a threat is perceived, there is an increase in cortisol production that leads to the activation of GR (Lupien et al., 2009). The high concentration of both MR and GR in the hippocampus suggests that the HPA-axis plays a role in learning and memory (Joels et al., 2012).

A complex relationship between the cortisol reactivity and neuropsychological functioning has been posited (Joels, 2006; Diamond et al., 2007). Studies have investigated the relationship between cortisol reactivity and neuropsychological functioning (Annett et al., 2005; Pruessner et al., 2007; Maldonado et al., 2008; Evans et al., 2012; Geoffroy et al., 2012; Ginty et al., 2012; Klaassen et al., 2013; Taverniers et al., 2013; Saridjan et al., 2014). Considerable variability exists in the assessment of cortisol (e.g., cortisol in saliva; urinary excretion of cortisol; cortisol in hair) and in the timing of these assessments (e.g., cortisol awakening response; evoked cortisol from a stimulus). There is, however, some research suggesting that child and adult cortisol reactivity to a stressor is highly similar (Kudielka et al., 2004; Yim et al., 2010).

Cortisol reactivity in children has typically been elicited with a stress task (e.g., Trier Social Stress Test) and neuropsychological functions, including executive functioning, then assessed. Extrapolating from available studies does suggest that very high levels of cortisol are implicated in poor performance on neuropsychological functioning (Quesada et al., 2012). Yet, little evidence exists for the portion of the inverted U-shape where modest cortisol reactivity is associated with increases in neuropsychological function. The present study seeks to address this gap in the literature.

In psychological research, cortisol production has been evaluated as area under the curve (AUC) and has commonly been used in laboratory settings as a metric for total cortisol production in response to a stimulus, such as the Trier Test. AUC can determined with respect to change over time, typically referred to as AUC with respect to ground (AUC*_G_*) or overall intensity of the cortisol response, referred to as AUC with respect to increase (AUC*_I_*)(Pruessner et al., 2003). Both measures inform the researcher about the cortisol response, yet can be problematic as individual baseline cortisol values can differ. Only recently have differences in cortisol measures been directly compared in children with findings indicating two principal components comprised of total cortisol production and change in cortisol over time (Khoury et al., 2015). A remaining challenge has been the ability to compare cortisol reactivity differences between individuals. We propose a unique cortisol reactivity measure that can be compared across individuals, what we have termed “normalized cortisol” response. Our approach comparable to AUC*_I_* as proposed by Pruessner et al. (2003), as both normalized cortisol and AUC*_I_* provide a measure of intensity of the response. Normalized cortisol is conceptually similar to AUC*_I_* in the value representing individual sensitivity, what we describe as “cortisol reactivity.” What normalized cortisol allows is a direct comparison between individuals on the change in cortisol response when two timepoints are used for analysis. Normalized cortisol has been previously utilized and found to be predictive of working memory (Tang et al., 2011) as well as behavior (Tang et al., 2012). Taken together, these studies suggest that cortisol reactivity can be related to neuropsychological functioning; however, translation of these models has been absent. The current study attempts to address this gap by examining both traditional and normalized measures of cortisol reactivity upon neuropsychological functioning in children with mild/moderate asthma. The aim of the current study is to test the contributions of traditional and normalized cortisol at study entry and at year 1 follow-up to subsequent child neuropsychological function.

Here previous work with data from an ancillary study convenience sample within the Childhood Asthma Management Program (CAMP) is presented. Our aim in this presentation is to examine the relationship between child cortisol reactivity to adrenocorticotropic hormone (ACTH) and neuropsychological function at year 3 of the CAMP study. More specifically, we examined cortisol reactivity at entry into CAMP and again 1 year later. CAMP was a multisite clinical trial investigating efficacy and safety of two treatments versus a placebo for children with mild to moderate asthma. CAMP utilized a multilevel approach to assess child health status, including neuropsychological and psychological functioning.

Two working hypotheses were developed to address the study aim. Our first hypothesis was that we predicted that cortisol measures (cortisol baseline and cortisol post-ACTH) at study entry and year 1 would predict children’s attention and memory function at year 3. Second, we hypothesized that individually normalized cortisol, what we call CORT_NORM–ACTH_, at study entry and year 1 could predict children’s year 3 attention and memory functioning.

## Materials and Methods

### Participants

Children ages 5–12 (*N* = 62 at study entry) participating in the CAMP randomized clinical trial for children with mild/moderate asthma were recruited and enrolled at two clinical centers (Albuquerque, NM and St. Louis, MO). The primary goal of the CAMP trial was to determine the long-term effects of three inhaled medications (budesonide, nedocromil, and placebo) for mild and moderate asthma in children. CAMP eligibility criteria included chronic asthma (asthma symptoms at least 2 times per week for 6 months, need for a bronchodilator at least 2 times per week, and daily use of an asthma medication. A 28-day asthma medication washout was part of the initial trial eligibility screening. A more complete description of the inclusion/exclusion criteria are reported (CAMP, 1999). CAMP participants were offered an option to participate in the HPA ancillary study and were enrolled in this prior to randomization (Bacharier et al., 2004). Participants in the HPA ancillary study had completed a 28-day drug washout when procedures described for this project were completed and before the randomization visit. Demographic and asthma severity information for ancillary study participants are presented in Table 1.

**TABLE 1:** Study participant characteristics at randomization and year 3.

	**Randomization**	**Year 3 Follow Up**
Number of participants	62	59
Age M(SD)	9 (2) yrs.	12 (2)
Range	5–13 years	8–15 years
Child Sex	40 Male	37 Male
	22 Female	22 Female
Child Ethnicity/Race	43 White	42 White
	11 Hispanic	9 Hispanic
	8 Other	8 Other
**Treatment Groups**		
Budesonide	17	17
Nedocromil	17	16
Placebo	28	26
**Asthma Severity**		
Mild	29	28
Moderate	33	31
**Pre-bronchodilator**		
FEV1% predicted ^∗^	94.23 (12.54)	94.88 (16.01)
FEV1/FVC ratio% ^∗^	78.79 (20.08)	76.59 (9.23)

*^∗^FEV1% predicted: Forced expiratory volume maximum in one second percent predicted. ^∗^FEV1/FVC ratio%: Forced expiratory volume in one second/Forced vital capacity (total volume of air expired).*

### Procedures

Procedures for the HPA ancillary study were reviewed and approved by the institutional review boards at the University of New Mexico Health Sciences Center and Washington University and entailed a separate consent from the CAMP trial. Written informed consent was obtained from the parents of the participants in this study. CAMP eligibility procedures have been previously described (CAMP, 1999) and consisted of 28-day medication washout prior to randomization. Cortisol reactivity and child neuropsychological functioning were assessed 2 weeks prior to randomization, hereafter referred to as study entry. Cortisol reactivity was again assessed at year 1 post randomization. At year 3 child neurobehavioral/neuropsychological functioning was assessed.

### Cortisol and ACTH Stimulation Procedure

Stimulation of adrenocorticotropic hormone (ACTH) is a highly controlled method for evoking cortisol reactivity and is considered a standard method for measuring cortisol responsiveness (Zollner et al., 2011). ACTH stimulation needs to be conducted in a controlled setting and requires that the participant be fasting for a minimum of 8 h and free of medications that can affect the HPA axis, including steroids for asthma exacerbation.

Data collection procedures for the HPA ancillary study were carried out within the General Clinical Research Center (GCRC) at each institution following a standard protocol (Bacharier et al., 2004). Children were admitted to the GCRC the night prior to the ACTH stimulation procedure and a parent was allowed to accompany the child if requested. Children were required to be in a fasting state (i.e., nothing by mouth after midnight). Baseline plasma cortisol (CORT_*BASELINE*_) was measured by circulating cortisol and assessed from a fasting morning blood draw from an intravenous line placed on the morning of the ACTH stimulation test. This was immediately followed by ACTH infusion, with serum cortisol examined at 30 min after ACTH infusion (CORT_POST–ACTH_).

On the morning of the procedure, a blood sample was drawn shortly after child awakening, between 7 and 9 am. Immediately following the blood draw, 0.25 mg of ACTH was administered over 60 to 90 s. Data collection included the date and time of the initial blood draw, time of ACTH infusion, time of 30-minute blood draw, and serum cortisol at baseline and 30 min after ACTH infusion. Samples were stored at −80°C until assayed at local laboratories using manufacturer procedures (Cortrosyn; Organon United States Inc., Bedford, OH, United States).

The current study examines plasma cortisol at baseline (CORT_BASELINE_), and uses two measures of 30-minute post stimulation cortisol. The first 30-minute post stimulation cortisol measure is the raw total concentration of cortisol (CORT_POST–A__CTH_) and the second is the raw total concentration of cortisol normalized by the baseline level of cortisol (CORT_NORM__–ACTH_ = (CORT_POST–A__CTH_ − CORT_BASELINE_)/CORT_BASELINE_ × 100). This measure is conceptually different from the CORT_POST–A__CTH_ measure as it provides a common measure for individual change in cortisol relative to the individual’s baseline.

The CORT_NORM__–ACTH_ measure used here differs from a “cortisol reactivity” measure (i.e., post-stress level−baseline level) and has been described (Tang et al., 2012). The CORT_NORM–ACTH_ allows for examination of whether the same amount of cortisol increase from baseline has a different impact on individual functioning depending upon the baseline concentration. We believe that stress regulation dynamics may be an important part of the brain’s hormonal signaling.

### Neuropsychological and Psychological Function

Neuropsychological functioning was assessed at two time points, study entry and year 3 (Annett et al., 2005). The current focus examines the temporal relationship of the cortisol reactivity at study entry and year 1 to child neuropsychological function at year 3. Procedures were standardized across CAMP settings through training, certification, and utilization of a CAMP-specific test administration and scoring manual (Childhood Asthma Management Program Behavioral Scientists Group, 1994). Order of test administration was maintained across settings (i.e., Wechsler Intelligence Scales: WPPSI-R/WISC-III; Woodcock- Johnson Psychoeducational Battery-Revised: WJ-R; Wide Range Assessment of Memory and Learning: WRAML; Gordon Diagnostic Systems: GDS).

Measures of children’s attention (GDS) and memory (WRAML) were ordered to standardize the delay interval for measurement of long-term verbal recall and visual recognition memory. WRAML Memory Screening Index subtests were administered followed by the Verbal Learning delayed recall subtest. This was followed by administration of three GDS subtests. WRAML Story Memory delayed recall and recognition subtests were then administered; thus, a standardized delay interval of approximately 30 min occurred.

Breaks in the testing session were provided based upon the judgment of the psychometrist. In order to maintain a standardized time interval for long-term recall and recognition, breaks were discouraged during this interval in the protocol. All neuropsychological data were obtained in a single session.

## Measures

### Wechsler Intelligence Scales (WPPSI-R/WISC-III) (Wechsler, 1989, 1991)

Similarities, Vocabulary and Block Design were administered and a deviation quotient calculated. This triad correlates adequately with long forms, *r*^2^ = 0.878 (Sattler, 1991). The WISC-III Symbol Search subtest was included when children were 6 years and older.

### Woodcock-Johnson Psychoeducational Battery–Revised (WJ-R) (Woodcock and Johnson, 1990)

Dictation, Letter-Word Identification, and Applied Problems, which comprise the Skills Cluster composite score, were used to evaluate academic achievement. Reliability coefficients were reported as 0.918, 0.938, and 0.915, respectively.

### Wide Range Assessment of Memory and Learning (WRAML) (Sheslow and Adams, 2003)

Wide Range Assessment of Memory and Learning memory screening subtests (Picture Memory, Design Memory, Verbal Learning and Story Memory) and Memory Screening Index were utilized. These subtests have alpha reliabilities ranging from 0.83 to 0.92. WRAML subtests are moderately related to one another, with inter-correlations of at least 0.25 (Sheslow and Adams, 2003).

### Gordon Diagnostic System (GDS) (Gordon and Mettelman, 1987)

Attention was assessed by the GDS, which is a self-contained/portable device. Delay, Vigilance and Distractibility tests were administered. Each task runs between 12 and 14 min. Test-retest reliability for each task has been reported: delay task (0.68); vigilance task (0.84); and vigilance (0.85).

### Data Analysis

**FIGURE 1:**
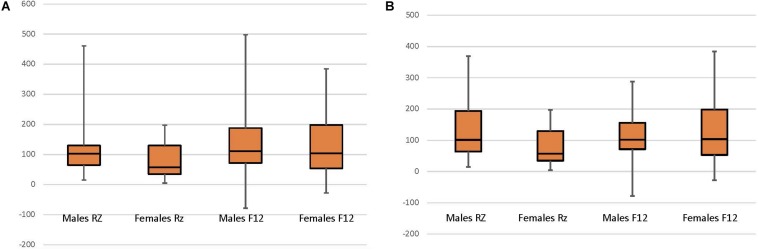
Child sex and normalized cortisol at CAMP randomization (RZ; *N* = 62) and 12-month follow up (F12; *N* = 58). **(A)** All data included. **(B)** Data with 5 extreme outliers (1.5 × the Interquartile Range) deleted, include the Male RZ (1 case) and Female RZ (4 cases).

Analyses were conducted using SPSS 25 (IBM Corp. Released 2017. IBM SPSS Statistics for Mac, Version 25 Armonk, NY: IBM Corp.). To determine cortisol reactivity as a stable individual variable, cortisol reactivity measures were correlated. The analyses utilized multiple linear regression to examine the relationship between cortisol values (at study entry and year 1) and year 3 child neuropsychological functioning (attention and working memory). To ensure assumptions for multiple linear regression modeling variables for analysis were examined for normality. Specifically, cortisol data were checked for normality and outliers were identified using bag plots (Rousseeuw et al., 1999). There were 5 extreme outliers (1.5 × the Interquartile Range) which were deleted from analyses (see Figure 1). Subsequent sensitivity analyses revealed no differences in findings between inclusion and exclusion of the 5 extreme outliers. Child neuropsychological variables exhibited finite variances and were normally distributed.

Covariates initially entered in the multiple linear regression analysis included child age at enrollment and sex. In order to determine whether there was an effect of CAMP treatment group on child cortisol (i.e., whether treatment group should be another covariate), *t*-tests between these variables found that, consistent with previous research findings (Bacharier et al., 2004), treatment condition was not associated with child cortisol reactivity and therefore not included in analyses. Independent variables subsequently entered included cortisol measures from study entry and year 1 (CORT_BASELINE_, CORT_POST–A__CTH_, and CORT_NORM_EVOKED_). Each cortisol measure was individually evaluated with multiple linear regressions, as they share substantial variance. Dependent neuropsychological variables included child attention (GDS Delay, Vigilance and Distractibility total correct) and memory (WRAML Memory Screening Index). Additionally, intelligence (WISC-III full scale IQ), and academic achievement (Woodcock-Johnson) variables (3 subtests and Skills Cluster composite) were tested in regression models to evaluate for possible confounding with the primary variables of interest.

Sex has been shown to influence child cortisol (Quinn et al., 2014), thus repeated measures ANOVA examined child sex and cortisol (CORT_BASELINE_ and CORT_NORM_POST–ACTH_) across the two collection periods (study entry and year 1). As noted above, five outliers were identified and dropped, resulting in a normal distribution of cortisol for analysis. Follow-up tests from this analysis were performed as necessary and included *t*-tests as well as ANOVAs to examine the effects of time and child sex in isolation.

## Results

Table 2 highlights associations among cortisol measures. Modest correlations are evident between entry and year 1 CORT_BASELINE_ (*r* = 0.431) as well as entry and year 1 CORT_POST–ACTH_ (*r* = 0.383). Noteworthy is the apparent individual stability of normalized cortisol from study entry to year 1: CORT_NORM_POST–ACTH_ *r* = 0.520. Further exploration of cortisol activity was accomplished by examining AUC with respect to ground for CORT and for CORT_NORM_, with findings indicating that neither value provides substantially different predictive value (CORT AUC = 0.521; 95% CI:0.416, 0.626); CORT_NORM_ AUC = 0.572; 95% CI:0.470, 0.675). Descriptive statistics from child neuropsychological variables are provided in Table 3.

**TABLE 2:** Correlation of cortisol measures.

	**Entry CORT_BASELINE_**	**Entry CORT_POST–ACTH_**	**Entry CORT_NORM–ACTH_**	**Year 1 CORT_BASELINE_**	**Year 1 CORT_POST–ACTH_**	**Year 1 CORT_NORM–ACTH_**
Entry CORT_BASELINE_	–					
Entry CORT_POST–ACTH_	0.553^∗∗^	–				
Entry CORT_NORM–ACTH_	–0.799^∗∗^	–0.199	–			
Year 1 CORT_BASELINE_	0.431^∗∗^	0.356^∗∗^	0.356^∗∗^	–		
Year 1 CORT_POST–ACTH_	–0.001	0.383^∗∗^	0.177	0.079	–	
Year 1 CORT_NORM–ACTH_	–0.441^∗∗^	–0.231	0.520^∗∗^	–0.799^∗∗^	0.178	–

*^∗∗^*p* < 0.01.*

**TABLE 3:** Child neuropsychological functioning at Randomization and Year 3.

	**Randomization**	**Year 3**
	
	**M (SD)**	
WISC-III/WPPSI-R ^∗^	108.0 (15.4)	104.6 (13.4)
**WRAML ^∗∗∗^**		
Picture Memory	10.9 (2.8)	10.3 (2.3)
Design Memory	10.3 (2.5)	9.9 (2.9)
Verbal Learning	11.5 (3.0)	11.42 (2.7)
Story Memory	11.2 (2.7)	10.4 (2.9)
**GDS (Total Correct) ^∗^**		
Delay	98.3 (12.7)	105.10 (11.2)
Vigilance	98.83 (16.3)	102.75 (17.4)
Distractibility	102.75 (19.5)	108.80 (15.6)
Woodcock-Johnson-III Skills Cluster ^∗^	105.23 (15.7)	101.15 (12.7)
**Child Behavior Checklist ^∗∗^**		
Competence	50.8 (8.7)	49.0 (9.0)
Externalizing	50.4 (9.7)	49.9 (8.5)
Internalizing	53.1 (9.2)	52.4 (9.9)
Withdrawn	53.7 (5.6)	54.5 (6.4)
Somatic	56.7 (7.2)	57.1 (7.3)
Anxious/Depressed	55.5 (6.8)	54.5 (6.2)
Child Depression Inventory (raw score)	5.1 (3.8)	4.0 (3.9)
Revised Children’s Manifest Anxiety Scale ^∗∗^	46.6 (13.6)	41.8 (11.2)

*^∗^Standard score (*M* = 100, *SD* = 15). ^∗∗^T-scores (*M* = 50, *SD* = 10). ^∗∗∗^Scaled scores (*M* = 10, *SD* = 3). WISC-III/WPPSI-R: Wechsler Intelligence Scale for Children–Third Edition/Wechsler Preschool and Primary Scale of Intelligence-Revised. WRAML: Wide Range Assessment of Memory and Learning. GDS: Gordon Diagnostic System.*

### Cortisol at Study Entry Predicts Year 3 Child Sustained Attention

Using multiple linear regression, we conducted analyses for each CORT measure, within initially entering control variables (child age and child sex) and in the second block child CORT measures. When examining the association of cortisol concentrations at study entry with neuropsychological functioning at year 3, we observed that CORT_BASELINE_ was associated with GDS Vigilance total correct score (sustained attention), and that child age was kept in the model [*F*(2, 57) = 4.614, *p* = 0.014, *R*^2^ = 0.144; Table 4]. There was also a significant association between CORT_NORM–ACTH_ and GDS Vigilance, for this particular model, and child sex was retained in the model (CORT_NORM–ACTH_ *F*(2,56) = 5.673, *p* = 0.006, *R*^2^ = 0.168; Table 4). CORT_BASELINE_ response was positively associated with GDS Vigilance, while CORT_NORM–ACTH_ response was negatively associated with GDS Vigilance. Thus, while both CORT_BASELINE_ and CORT_NORM–ACTH_ are highly correlated [*r*(62) = −0.799, *p* = 0.001], each contributes to children’s sustained attention. The relative rise in cortisol represented in CORT_NORM–ACTH_ does appear to explain a slightly greater proportion of children’s sustained attention skills. In contrast, cortisol reactivity was not associated with the WRAML Memory Screening Index scores. Similarly, child IQ and academic achievement was not predicted by cortisol at study entry.

**TABLE 4:** Cortisol predicts child sustained attention and memory at Year 3.

		**Unstandardized**		**Standardized**			
		**Coefficients**		**Coefficients**			
		**B**	**Standard Error**	**Beta**	***t***	**Significant**	***R*^2^**
**Year 3 GDS Vigilance (Sustained Attention)**							
CORT response variables at Entry	Age at Enrollment	2.797	1.115	0.316	2.509	0.015	0.144
	CORT_BASELINE_	0.73	0.361	0.254	2.017	0.049	
	
	Sex	6.539	4.509	0.186	1.450	0.153	0.168
	CORT_NORM_ACTH_	−0.061	0.025	−0.312	−2.433	0.018	

CORT response variables at year 1	Age at Enrollment	3.368	1.227	0.342	2.745	0.008	0.198
	CORT_NORM_ACTH_	−0.047	0.019	−0.311	−2.497	0.016	

**Year 3 WRAML Memory Screening Index**							
CORT response variables at year 1	CORT_POST–ACTH_	0.666	0.248	0.346	2.687	0.010	0.120

### Cortisol at Year 1 Predicts Child Sustained Attention and Memory

Similar to the previous set of analyses, additional analysis examined the association of cortisol reactivity at year 1, and child attention and memory at year 3. Multiple linear regression analyses were conducted for each cortisol measure, in the first block control variables (child sex and child age) and in the second block child CORT response variables were entered. CORT_NORM__–ACTH_ and CORT_POST–A__CTH_ at year 1 affected different aspects of neuropsychological functioning. Year 1 CORT_N__ORM–ACTH_ and age were significantly associated with year 3 GDS Vigilance [*F*(2,52) = 6.41, *p* = 0.003, *R*^2^ = 0.198; Table 4]. Child IQ and academic achievement was not predicted by year 1 cortisol. Thus, both study entry and year 1 CORT_NORM__–ACTH_ predicted year 3 sustained attentions. In addition, CORT_POST–A__CTH_ predicted children’s memory performance [WRAML Screening Index; *F*(1,54) = 7.222, *p* = 0.010, *R*^2^ = 0.120; Table 4]. If CORT_*ACTH*_ was elevated at year 1, WRAML Memory Screening Index scores were elevated 2 years later. Subsequent analyses revealed that the variance in memory screening scores was related to both non-verbal memory tasks that comprise the index score: Picture Memory [*F*(1,54) = 5.198, *p* = 0.027, *R*^2^ = 0.089], Design Memory [*F*(1,54) = 4.880, *p* = 0.032, *R*^2^ = 0.084]. In summary, year 1 normalized cortisol explained a small proportion of children’s attention (approximately 20%) and CORT_POST–ACTH_ predicted memory (approximately 12%) functioning.

In order to test whether CORT_BASELINE_ and CORT_NORM–ACTH_ vary by sex and time, two repeated measures ANOVAs evaluated the interactive effect between child sex and time of cortisol collection (study entry, year 1) upon cortisol reactivity. For CORT_BASELINE,_ no interaction [*F*(1,56) = 1.813, *p* = 0.184] or time [*F*(1,56) = 0.085, *p* = 0.772] effect was observed, however, a significant sex effect was observed [*F*(1,56) = 6.736, *p* = 0.011]. Follow-up tests show that females had higher CORT_BASELINE_ than males. For CORT_NORM–ACTH,_ an interaction effect was observed [*F*(1,53) = 2.711, *p* = 0.106] as well as main effects for time [*F*(1,53) = 8.928, *p* = 0.004] and child sex [*F*(1,53) = 3.477, *p* = 0.068]. Follow-up *t*-tests examined the effect of time on cortisol for females and males across the 2 time periods. For females, there was a significant difference in cortisol between study entry and year 1 [*t*(18) = 2.382, *p* = 0.028], with CORT_NORM–ACTH_ increasing between study entry than year 1. No difference was found for males [*t*(35) = 1.327, *p* = 0.193] (Figure 1). Follow-up tests examining the effect of sex revealed a significant difference between male and female cortisol at study entry [*F*(1,60) = 6.399, *p* = 0.014] but not at year 1 [*F*(1,58) = 0.225, *p* = 0.637), with females having lower CORT_NORM–ACTH_ at study entry and no difference in cortisol between males and females at year 1.

In summary, year 1 CORT response measures predicted small proportions of children’s sustained attention and memory skills assessed at year 3. The relative rise in cortisol reactivity (CORT_NORM–ACTH_) appears to explain slightly greater amount of the variances observed at year 3 (*R*^2^ 0.168 and 0.198, respectively), though these are relatively small amounts of the overall variance structure.

## Discussion

In a longitudinal study of children with mild to moderate asthma, we have identified two new contributions to the science of HPA axis functioning. First, a predictive relationship of an initial cortisol value (CORT_BASELINE_) and an alternative cortisol reactivity (CORT_NORM–ACTH_) and subsequent child neuropsychological functioning over 3 years was observed. Children’s cortisol reactivity for this study was elicited with ACTH using a standardized protocol in a hospital setting, thus was substantially different from eliciting a stress response with psychologically stressful tasks (e.g., Trier social stress task). The findings presented provide data on the biological reactivity of cortisol absent a psychological stress stimulus. We suggest that our findings represent a limited description of how the HPA axis responds to provocation by ACTH, yet how this response functions for individuals in a real-world setting using a psychological stress stimulus cannot be simply assumed. Our work represents an extension of earlier findings related to the cortisol response and long-term child neuropsychological functioning with the exploration of an alternative measure of cortisol reactivity (CORT_NORM–ACTH_).

The findings presented raise the possibility that the alternative measure of cortisol reactivity (CORT_NORM–ACTH_) is relatively stable over the course of 1 year (*r* = 0.520) and may be utilized to predict subsequent child neuropsychological functioning, albeit a small proportion. The alternative measure of cortisol used is similar to that of Pruessner’s cortisol response intensity and allows for greater opportunity to compare cortisol responses between individuals, much in the manner that standardized scores allow for between individual comparisons of neuropsychological functioning. Our findings reveal that children with higher CORT_BASELINE_ and lower CORT_NORM–ACTH_, at study entry exhibited higher sustained attention 3 years later. Lower CORT_NORM–ACTH_ at study entry and year 1 was also related to higher sustained attention 2 years later. Additionally, higher CORT_POST–A__CTH_ at year 1 was related to increased non-verbal memory 2 years later. These findings support our hypothesis that cortisol reactivity can predict later neuropsychological abilities among school age children. Furthermore, CORT_NORM–ACTH_ is influenced by child sex, with this influence occurring for females, as evident in the rise in relative cortisol reactivity from study entry to year 1. Females demonstrated higher cortisol reactivity than males at study entry, yet this difference did not persist to the year 1 follow up.

Correlations between child cortisol and neuropsychological functioning variables were statistically significant for the CORT_NORM–ACTH_ measure at multiple time points, however, other cortisol measures were less predictive, suggesting that CORT_NORM–ACTH_ to be a more stable measure of cortisol reactivity in the absence of a stressor task.

### Evoked Cortisol as a Predictive Measure, Should Be Utilized More Frequently

We assessed three distinct measures of cortisol to discern which aspect of cortisol reactivity could be most predictive of later neuropsychological abilities in this convenience sample of children. These 3 methods included: CORT_BASELINE_, a measure of cortisol concentrations before evoking a cortisol reactivity via ACTH stimulation; CORT_POST–A__CTH,_ a raw measure of cortisol concentration; and CORT_NORM–ACTH_, a normalized 30-minute post stimulation cortisol measure which evaluates the relative percentage rise in cortisol from baseline to 30 min post ACTH stimulation. Past literature has employed different methods to measure cortisol production, but to our knowledge this is one of the first studies, using the same sample population to compare the predictive ability of these different cortisol reactivity measures. Comparing the predictive power of multiple cortisol measures can help elucidate how future studies may choose to assess cortisol when exploring its relations to neuropsychological function.

The normalized evoked cortisol measure of cortisol reactivity was a robust and predictive measure. CORT_NORM–ACTH_, which is the percentage change in cortisol from baseline, has not been frequently utilized in human subjects, but has been used previously in animal studies assessing the relationship between corticosterone across generations, corticosterone and behavioral inhibition, as well as corticosterone and working memory (Tang et al., 2012; Dinces et al., 2014). This method was unique in using normalized evoked cortisol to achieve higher predictive power for behavioral inhibition and working memory (Tang et al., 2013). Normalized evoked cortisol may provide a stable individual cortisol reactivity and the increased use of this specific cortisol measure merits additional exploration in future studies.

The predictive ability of the normalized evoked cortisol measure changed from study entry to the year 1 follow-up, with an increased association between child cortisol reactivity and attention observed with cortisol at study entry compared to 1 year follow up. In attempting to determine what might account for this change in predictive power, child sex differences in cortisol and variance were examined. Interestingly, female CORT_NORM–ACTH_ levels increased by an average of 58.7% while male CORT_NORM–ACTH_ only increased by 16.8%. There are multiple interpretations for why this may be: children, particularly female children, entering into puberty or pre-puberty may have increases in their cortisol (Stroud et al., 2004, 2011; Shirtcliff et al., 2012) or the treatments given to the children to control their asthma might have sex dependent effects. These results highlight the need to carefully study child HPA function and possibly even neuropsychological functioning in a sex dependent manner and for studies to be designed considering sex differences. This finding extends the similar finding reported by Annett et al. (2005), as it assesses cortisol at two time points a year apart. Additionally, this observation of females having higher baseline responses but lower evoked responses (at study entry), provides confirmation for the finding that when baseline response levels are high, the same individual’s evoked levels will be relatively low (Levine, 1960).

### Cortisol Can Predict Later Neuropsychological Functioning

Our results indicate that HPA reactivity, as measured through cortisol, can predict later neuropsychological functioning of children with mild to moderate asthma. Additionally, this well characterized sample demonstrated relatively normal levels of intelligence, neuropsychological functioning and psychosocial adjustment over the 3 years of study. Our finding supports previous research that found cortisol can predict verbal memory abilities 5 years later (Geoffroy et al., 2012). Moreover, these findings highlight the relationship between moderate cortisol and increased neuropsychological functioning (Sandi and Pinelo-Nava, 2007). We have previously demonstrated that when analyzing data collected at study entry, child working memory was positively associated with overall cortisol reactivity in response to ACTH stimulation (Annett et al., 2005). Findings from the current study extend our previous work within a longitudinal framework of a carefully characterized group of children. Within this context, cortisol reactivity was differentially predictive of later neuropsychological functioning. Our findings support this hypothesis because CORT_BASELINE_ and CORT_NORM–ACTH_, two opposing measures related to modulation of the stress response, were both related to sustained attention, while CORT_POST–A__CTH_, a measure of raw cortisol production was only associated with non-verbal memory. Cortisol concentrations are not uniform across brain functions and it may be that our initial (CORT_BASELINE_) and alternative (CORT_NORM–ACTH_) measure of cortisol production are demonstrating differential sensitivity to related brain functions when examined over time. Additional translational work may provide further elucidation of the mechanism of action.

The influence of cortisol on child neuropsychological functioning may occur with GR activation and associated GR density in the hippocampus. In animal studies, both environmental enrichment and brief exposure to a moderate repeated stressor leads to enhanced GR expression and density as well as spatial working memory performance (Sampedro-Piquero et al., 2014). In humans, administration of MR antagonists or GR agonists leads to impaired memory performance, indicating that to improve memory performance, high activation of the MR and low activation of the GR is preferred (Groch et al., 2013; Rimmele et al., 2013). These human studies concur with animal findings, as when there is a higher capacity of GR, then proportionally, the same amount of cortisol will account for less occupancy, which is needed for enhanced memory performance. Thus, both animal and human studies imply the mechanism by which cognition may be affected by cortisol could occur through differential activation and balance between the glucocorticoid receptors (de Kloet, 2014).

## Implications

This study suggests that among children, like adults, cortisol reactivity as measured through cortisol production levels, can predict later neuropsychological abilities. Neuropsychological performance may be affected by elevated basal and lower evoked cortisol, yet using normalized cortisol may mitigate the impact of individual variability. The findings from the current study need corroboration from future research. Utilizing cortisol to benchmark the stress response may have implications for improving children’s future neuropsychological functioning by directing interventions that directly improve the child’s management of stressful events. For example, school based mindfulness may help to improve children’s attention skills (Crescentini et al., 2016).

The importance of examining normalized evoked cortisol as a measure of cortisol reactivity and that this HPA measure may have clinical utility to predict child future attention a central finding. Additionally, our results suggest the robustness and higher predictive power of normalized cortisol, given that this leads to significant findings across time points, unlike other cortisol measures. In addition, normalized cortisol uniquely adds to the way in which cortisol is measured because rather than providing a rough amount or proportion of cortisol secreted, it reports the percentage of change in cortisol secondary to a stressor. This study highlights that the specific method for measuring cortisol can affect findings for relationships of cortisol with outcome variables. Specifically, we recommend additional testing of normalized evoked cortisol in the future in order to evaluate its veracity in other contexts.

## Limitations

In this study, cortisol was uniquely collected from a convenience sample who underwent ACTH stimulation testing rather than a real-world exposure to a psychological stressor. Cortisol reactivity was sampled from plasma collected from both blood draws and an IV and it is unknown to what extent these procedures may have resulted in a stress response. Moreover, how the observed cortisol reactivity corresponds with a cortisol reactivity to a psychosocial stressor is unknown. The values of the cortisol samples may be confounded by the cortisol awakening response, in which cortisol rapidly increases for approximately 30 min after awakening. Despite the highly controlled setting, the exact time of child awakening and time of sample collection went unrecorded, allowing for speculation as to the contribution of the awakening response to the cortisol reactivity. Finally, exposure to adverse experiences (e.g., trauma, parenting quality) prior to trial entry was not assessed among participants. Extent of early life adversity has been recognized as contributing to neuroendocrine response and child neuropsychological functioning, though assessment of child emotion, child neuropsychological functioning and parent report of behavior suggest that participants demonstrated typical patterns of development. Given these limitations, future research should explore the relationship of HPA function with neuropsychological outcomes in a non-asthma sample and with less invasive procedures for measuring cortisol and with careful mapping of time of child awakening and cortisol collection.

## Concluding Statement

The objective of this presentation was to examine if HPA axis functioning as measured by cortisol production was associated with future neuropsychological functioning among a well-characterized group of children. As expected, in a sample of children with mild/moderate asthma normalized evoked cortisol was associated with neuropsychological functioning 2 and 3 years later. HPA dysregulation may impact future neuropsychological performance. This study highlights that the measure utilized for evaluating cortisol greatly affects derived findings and relationships with the specific outcome variables. Findings presented suggest the use of normalized cortisol in future studies as this measure may provide improved predictive ability for child developmental outcomes which could, in turn, have greater clinical utility than other measures of HPA function.

## Data Availability Statement

The datasets generated for this study are available on request to the corresponding author.

## Ethics Statement

Procedures for the HPA ancillary study were reviewed and approved by the local institutional review boards and entailed a separate consent from the CAMP trial.

## Author Contributions

RA designed and supervised the study. SD wrote the first draft. AT contributed to the methods and implementation of the normalized cortisol response. SD, LR, JB, and SH contributed to the writing and editing of the manuscript.

## Conflict of Interest

The authors declare that the research was conducted in the absence of any commercial or financial relationships that could be construed as a potential conflict of interest.
